# Anthracene Fibers Grown in a Microstructured Optical Fiber for X-ray Detection

**DOI:** 10.3390/ma7096291

**Published:** 2014-09-03

**Authors:** Stanton DeHaven, Russell Wincheski, Sacharia Albin

**Affiliations:** 1 NASA Langley Research Center, Hampton, VA 23681, USA; E-Mail: russell.a.wincheski@nasa.gov; 2 Department of Engineering, and Center for Biotechnology and Biomedical Sciences, Norfolk State University, Norfolk, VA 23504, USA; E-Mail: salbin@nsu.edu

**Keywords:** growth from melt, organic compounds, scintillating materials, optical fiber devices

## Abstract

Anthracene fibers are grown inside a microstructured quartz matrix to form a multicore optical fiber for X-ray detection. A modified fiber growth method for single crystal anthracene from the melt via the Bridgman-Stockbarger technique is presented. The anthracene fiber is characterized by using spectrophotometry, Raman spectroscopy, and X-ray diffraction. These results show the anthracene grown in fiber has high purity and a crystal structure similar to anthracene grown from liquid, vapor, and melt techniques. As an X-ray detector, the output is 12%–16% efficient between the energy ranges of 40 and 10 keV. The effect of materials and fiber processing are discussed.

## 1. Introduction

Anthracene is an organic semiconductor material that is used as a scintillator for ionizing radiation detection [1,2,3,4]. Its electroluminescent properties have been exploited as a base material for organic LEDs [5,6,7,8]. Anthracene has also been investigated as a sensor material for magnetic fields and pressure. Spectral fluorescence variations and quenching have been reported from magnetic field interactions with anthracene crystals [9,10,11]. Pressure applied to anthracene crystals affects the optical absorption and fluorescence spectra [12,13]. The light output of anthracene crystals is the highest of all organic scintillators with a decay time of approximately 32 ns [1,2,3].

The addition of quantum dots, carbon nanotubes, chemical coatings and liquids into microstructured optical fiber (MOF) provides various sensors for temperature, chemical, and X-ray detection [14,15,16,17,18,19,20]. These filled MOF devices operate based on various principles including fluorescence, absorption, and refraction index variations. Growing single crystal anthracene in quartz MOF allows utilization of the physical sensing properties of anthracene in an optical fiber.

The growth of single anthracene crystals has been extensively studied and is still a subject of research. Many experimental techniques have been explored to grow and characterize anthracene crystals [21,22,23,24]. Large anthracene crystals are typically grown from the melt in glass tubes using a modified vertical Bridgman-Stockbarger technique at very low growth rates [21]. The glass is subsequently etched away using HF acid to free the anthracene crystals.

These large single crystals are typically used in scintillation applications but are fragile. Smaller anthracene crystals grown from solution and vapor become part of electro-optic and non-linear optical devices. The deposition of anthracene crystals allows organic semiconductors for electronic applications such as field effect transistors [22,23,24].

The single crystal growth method for large diameter anthracene from the melt conditions was found to be unsatisfactory for fibers of micron-size diameters. Previous work on fiber growth focused on single core organic crystal fibers suitable for second-order harmonic generation [25,26]. This paper presents improvements in multicore organic single crystal anthracene fibers of a few microns in diameter for X-ray detection.

The anthracene fibers are characterized using spectrophotometry, Raman spectroscopy, and X-ray diffraction. The MOF presented can guide light in both the anthracene and quartz microstructure, allowing more scintillation photons to be guided by the fiber compared to when filling the MOF with anthracene. Improvements to the crystal growth and fiber configuration provide higher measured X-ray detection efficiency than the maximum theoretical output when the fiber is configured as a filled MOF [20].

We have successfully grown anthracene fibers of up to 12 cm long inside the MOF. The benefits of anthracene fibers integrated with the quartz MOF include increased photon collection, ease of handling due to the quartz support, enhanced functional reliability and flaw tolerance from multiple fibers in the quartz.

## 2. Experimental Section

**Figure 1:**
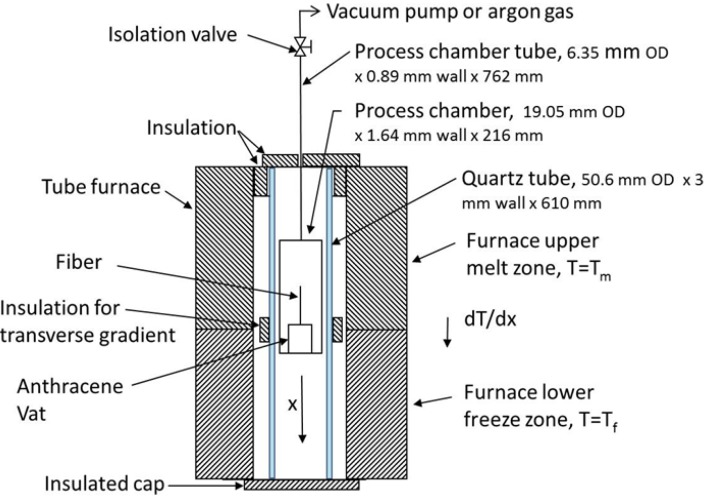
A schematic diagram of the furnace and chamber cross section of the vertical Bridgman-Stockbarger technique for growing single anthracene crystals inside microstructured quartz.

Figure 1 shows the schematic diagram of the experimental arrangement for growing single crystal anthracene in the quartz MOF. The tube furnace is separated into two zones, an upper 406 mm section for melting and a lower 203 mm section for freezing. The upper zone uses a higher temperature for thermal stability. The typical zone temperatures are 230 °C for the upper (T_m_) and 70 °C for the lower (T_f_) measured using K-type thermocouples. The tube furnace and controller regulates temperature to ±1 °C. A quartz tube is placed between the furnace heating elements and the chamber to enhance thermal stability. Segmented insulation on the outside of the quartz tube between high and low temperature zones provides a transverse temperature gradient.

The process chamber is constructed of 316 stainless steel tubing. This chamber holds the vat with fiber and allows process atmosphere control. The vat is constructed from a capped brass fitting 3 mm in diameter and 8 mm deep and acts as a large reservoir holding hundreds of times the amount of anthracene used in the quartz MOF. The quartz MOF extends to the bottom where capillary action fills the MOF when the anthracene melts. The anthracene used for the melt is >99% pure scintillation grade with no additional purification.

The vat is initially filled with anthracene and sealed and the quartz MOF is inserted through a small hole in the cap. The MOF is sealed to the cap and the assembly placed in the process chamber, which is then placed in the furnace and evacuated between 2 × 10^−2^ and 5 × 10^−2^ Torr for 48 h. Before energizing the furnace, 1.14 atm of high purity argon is added to the chamber.

The chamber is initially placed in the upper (melt) zone of the furnace; its temperature rises with the furnace temperature and is held at the 230 °C for one hour to melt the anthracene and allow the capillary action to fill the MOF.

Subsequently, the chamber translation begins and proceeds until a fixed distance is completed. After processing, the chamber is allowed to cool before removing the MOF. Visual inspection is then performed with an optical microscope incorporating side illumination from a 385 nm UV light source. After growing anthracene in the MOF, aluminum or PTFE is applied on the exterior via sputtering to investigate photon collection with these coatings and the effect on fiber handling.

The aluminum coating on the MOF was applied using DC magnetron sputtering. The argon pressure was 20 × 10^−3^ Torr and the power was 350 watts. Approximately 0.1 micron thickness was applied of 99.99% pure aluminum. 

The PTFE coating was applied using RF sputtering. An initial argon pressure between 5 × 10^−3^ and 20 × 10^−3^ Torr was applied at 250 watts power to initiate a glow. The argon flow was reduced and then shut-off after several minutes once the glow stabilized [27]. Approximately 0.1 micron thickness was applied of ASTM D1710 PTFE.

## 3. Results and Discussion

**Figure 2:**
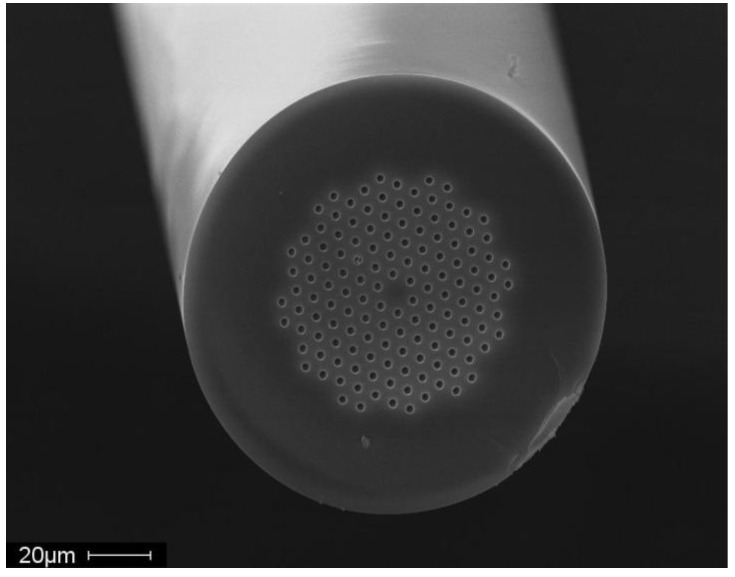
Microstructured quartz optical fiber cross-section SEM image taken at 30 keV. The fiber diameter is 125 microns with 138 air filled inclusions of 2.5 microns each. These inclusions become filled with anthracene.

An image of the quartz MOF used in our experiment is shown in Figure 2 and contains 138 air inclusions of 2.5 microns in diameter. After growth, the fiber has ~1 microgram/cm of anthracene inside the quartz MOF calculated from the fiber geometry and solid anthracene density of 1.25 g/cm^3^. The growth experiments from the melt were conducted using a modified Bridgman-Stockbarger technique with a stainless steel chamber holding the quartz fiber, anthracene, and argon gas blanket during processing as in our previous work [28]. This chamber was positioned in a tube furnace that provided a temperature gradient similar to the method described in [25]. A sealed vat supports the MOF fiber and provides a molten pool of anthracene where capillary action fills the quartz from the pool; a stainless steel process chamber allows vacuum and back-filling with high purity argon; an outer quartz tube with insulation induces a radial temperature gradient between the furnace and process chamber.

The Bridgman-Stockbarger technique uses a controlled temperature gradient for crystal growth from the melt at a minimum free energy state. The temperature gradient is longitudinal along the crystal growth direction. In the case of large crystal growth, the heat transfer occurs primarily through the anthracene. However, the microstructured quartz containing anthracene has the primary heat transfer path occurring through the quartz due to the relative size of the anthracene to the quartz and the higher thermal conductivity of quartz.

The approach to determine the range of speeds and the temperature gradient were based on experimental observations for void-free anthracene fiber. The chamber translated at a uniform speed which varied from 40 down to 3 mm/h. The lower speeds were typical for large crystal growth having a smaller temperature gradient [21].

Initially, the growth conditions for large crystal anthracene were tried but failed to grow the anthracene fibers. One explanation may be that the extremely low mass of both the microstructured anthracene and quartz require highly accurate temperature regulation for low undercooling growth; or perhaps the small amount of surface area and material in the fiber requires an extremely slow (<<1 mm/h) growth rate.

**Figure 3:**
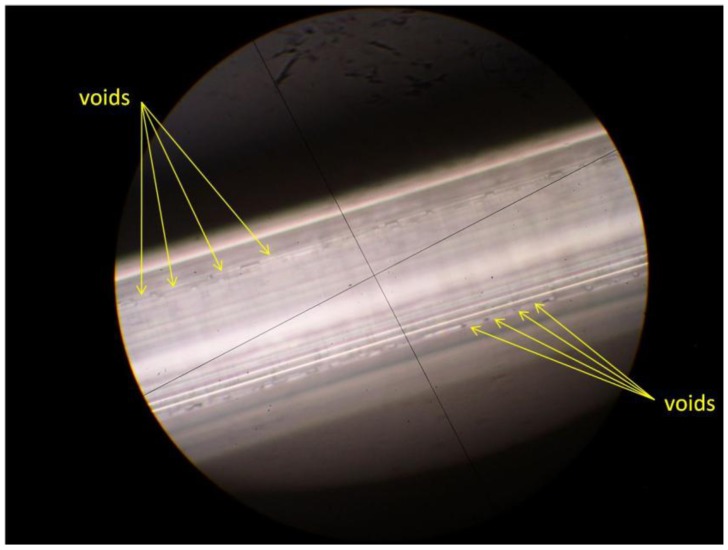
View of anthracene fibers in MOF at 500× showing voids along growth direction. Anthracene fibers grown without voids are translucent and not visible using incandescent light.

Speeds from 20 to 40 mm/h with upper and lower zones of 230 and 70 °C, respectively, produced the best fiber with few voids and high optical clarity. Experimental results showed two speed variations produced no benefit, nor did speeds over 40 mm/h or very low translation speeds. The low speed ranges considered in previous work did not produce continuous lengths of anthracene fibers [25]. Additionally, no satisfactory results were found at translation speeds below 15 mm/h using high or low temperature gradients. Figure 3 shows an image of anthracene fiber using incandescent light with voids along the growth direction. A high quality anthracene fiber is translucent and UV light is required to assess if voids exist by looking for scattering bright spots from defects.

**Figure 4:**
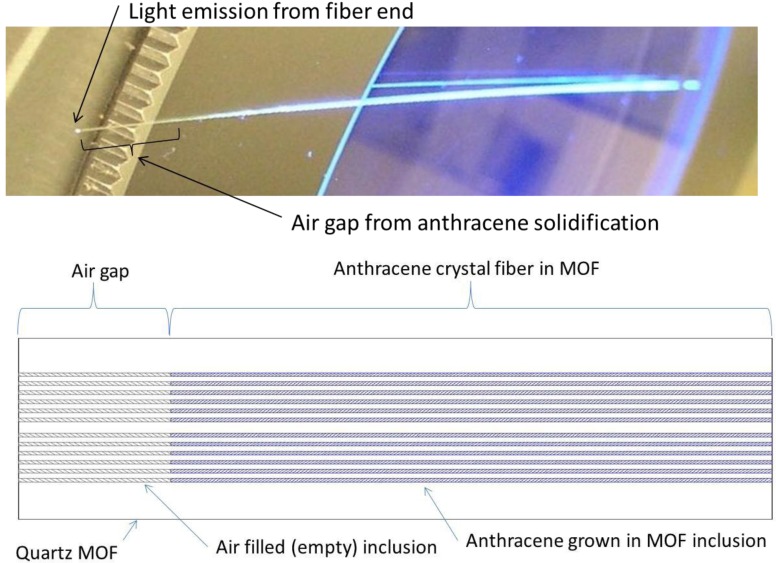
Anthracene in MOF: shown on a microscope stage illuminated with 385 nm UV light showing the air gap formed from solidification during the growth process and blue (420 nm) light from the fiber end; cross section diagram showing air gap at emission end.

The chamber material has a direct effect on void formation, and when constructed from higher thermal conductivity, materials did not yield anthracene crystals of lengths longer than 1 mm. Using a lower conductivity stainless steel chamber reliably and repeatedly produced approximately 12 cm continuous fibers. High quality anthracene fiber grown in the MOF is shown using visible and 385 nm UV light in Figure 4. During anthracene fiber growth, an air gap is formed at one end of the MOF from contraction due to solidification of anthracene. This effect occurs due to the higher density of solid anthracene compared to liquid and plays a role in light guidance and the X-ray detection efficiency of the fiber. Anthracene filled MOF is formed by removing the air gap section.

### 3.1. Material Characterization: Fluorescence Spectra

**Figure 5:**
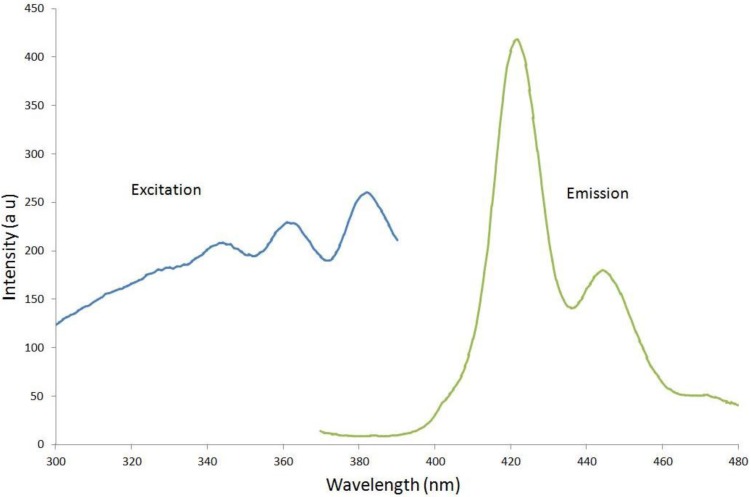
Excitation and emission spectra are shown from left to right for anthracene fibers in quartz. The emission spectral profile agrees with the data for microcrystalline anthracene from Ref [29].

The anthracene was characterized using spectrophotometry, Raman spectroscopy, and X-ray diffraction. The results were compared with those for solid crystalline anthracene data. Anthracene MOF excitation and emission data taken using a Hitachi F4500 luminescence spectrophotometer is shown in Figure 5. A 3-cm long fiber was centered in a fluorescence quartz cuvette suitable for the spectrophotometer.

The excitation spectrum was collected for a fixed emission wavelength of 420 nm and the emission spectrum was collected for a fixed excitation wavelength of 350 nm. The excitation spectrum shows very good agreement with previous crystal anthracene data [12,29]. The spectral peak and shape of the emission curve with a peak at 420 nm and a secondary peak at 445 nm compare well with the relative amplitude of microcrystalline anthracene published data [29]. 

#### 3.1.1. Material Characterization: Raman Spectroscopy

A Kaiser optical Raman RxN microscope system was used to collect the Raman spectral data from the MOF end. An optical microscope incorporated into the Raman spectrometer allowed selecting the locations for excitation where data was collected using a 785-nm wavelength laser and compared with data for crystalline anthracene [30,31,32,33,34]. Two orthogonal polarization angles of the laser were used, both in the plane of the MOF end. However, the polarization angle had little effect on the Raman modes and resultant spectra. The lack of laser polarization sensitivity is attributed to the lack of preferred orientation of the anthracene in the MOF end.

**Figure 6:**
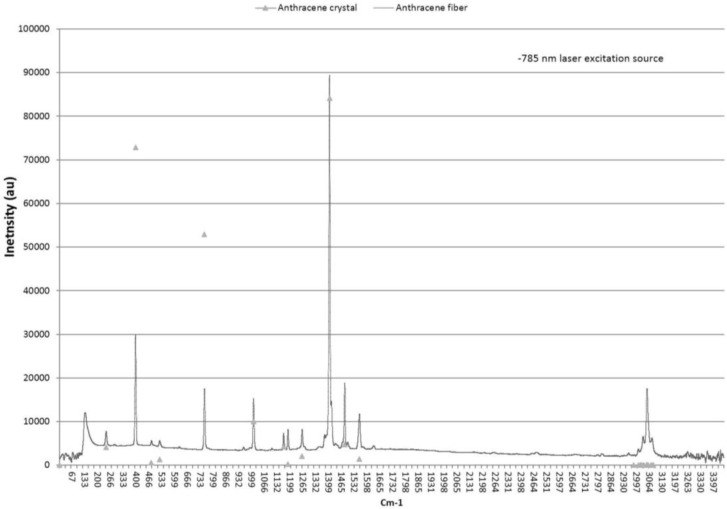
Raman spectra from anthracene fiber in the quartz matrix. Fiber data has ±1 cm^−1^ agreement with anthracene crystal data spectrum (indicated by triangular markers) [30].

Figure 6 shows the Raman spectra with the scaled anthracene crystal data from Table 1 of reference [30] for comparison. These peaks correspond to scattering associated with vibrational modes of the molecular solid [34]. The wavenumbers agree within ±1 cm^−1^. The amplitude of the predominant peak at 1403 cm^−1^ was scaled to that of reference [30] with the result that 395 and 753 cm^−1^ peaks have attenuated amplitude as compared to data in [30]. The relative amplitude is the same; the peak at 395 cm^−1^ is the second highest with the third highest at 753 cm^−1^. The 395, 753, 1007, 1403, 1482, and 1557 cm^−1^ wavenumber peaks are the highest amplitude corresponding to the a_g_ free molecular (D_2h_) Raman active symmetry and agree with theoretical wavenumber calculations and previous experimental relative intensity measurements [30,33]. These data demonstrate that the anthracene fiber has high purity and the molecular structure is similar to that of anthracene crystal grown from solution.

#### 3.1.2. Material Characterization: X-ray Diffraction

**Figure 7:**
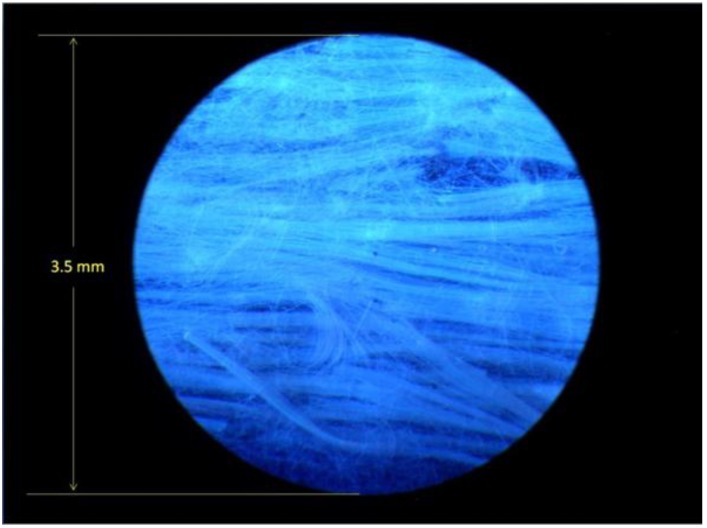
Anthracene fibers extracted from the quartz cladding and arranged as a thin film for XRD surface analysis at 50× magnification under UV light. The 2.5 micron diameter fibers form a yarn of anthracene fibers.

A Panalytical X-ray diffractometer was used to collect the X-ray diffraction (XRD) data. The anthracene fibers were released from the quartz MOF by etching in HF acid. Approximately 60 pieces of MOF fiber 1-cm in length were collected to make the XRD sample. The bare anthracene fibers were placed on a (100) silicon wafer in a rectangular pattern to simulate a thin film for surface analysis and a 10 mm × 3 mm sample was made for the diffractometer [24,35]. Figure 7 shows an image of the anthracene fibers at 50× magnification and illuminated with 385 nm UV light to produce blue (420 nm) florescence emission.

**Figure 8:**
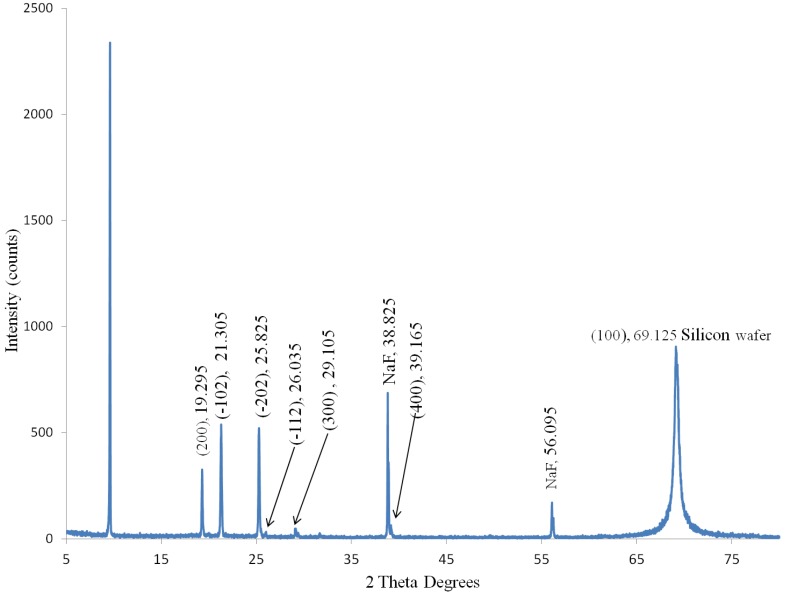
Anthracene X-ray diffraction (XRD) from bare anthracene fibers etched from quartz on a (100) silicon wafer. The (100) anthracene peak at 9.625 2 Theta degrees corresponds to the growth direction. Diffraction angles for peaks all correlate to the anthracene XRD card data [36], sodium fluoride [37], and silicon. The crystal orientation was taken from the ICCD data file [36].

Figure 8 shows the peaks from the anthracene X-ray diffraction scan. The XRD data for anthracene has excellent spectral agreement with the powder XRD data file for crystalline anthracene within ±0.04 degrees. The peak amplitudes in Figure 8 differ from the powder data due to the preferential orientation of the anthracene; however, the peaks at 19.295, 21.305, and 25.285 2θ angles with respective Miller indices of (100), (−102), (−202) agree with ICCD powder diffraction data [36]. Also shown are the peaks from NaF resulting from the HF acid neutralized by NaOH and the (100) peak from the silicon wafer [37].

The anthracene fiber has the same crystalline structure of the bulk powder as shown by Bragg diffraction angles. The main (100) geometric orientation peak is in the growth direction and is consistent with XRD data from high purity anthracene crystals grown from solution and the vapor phase [23,24]. It can be calculated using these Bragg angles that *d*_100_ = 9.18 Å, which is less than the single crystal powder diffraction length (*d*_100_ = 9.21 Å, P21/c) and solution growth crystal length (*d*_001_ = 9.23 Å, P21/a) but larger than the vapor phase length (*d*_001_ = 9.14 Å, P21/a) [23,24,36]. It is also noted that the Miller indices in Figure 8 for single crystal data use the P21/c space group orientation. However, most anthracene literature uses the P21/a space group notation [22,23,24]. The two space groups are equivalent for the anthracene monoclinic crystal [38].

### 3.2. X-ray Efficiency

**Figure 9:**
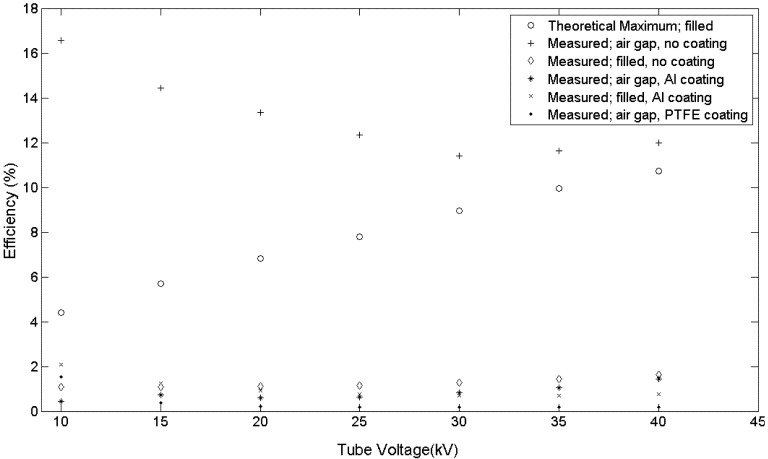
Anthracene increased efficiency from improved crystal growth and fiber configuration. The effect of coatings on fiber efficiency shows aluminum and PTFE thin films yield near the same efficiency with or without an air gap.

The efficiency was measured using the setup and procedure previously described for liquid filled optical fiber and the results are compared with those from previous anthracene fiber efficiency [20,28]. Here, a 40 kV, 4 W tube was the source with a photomultiplier photon counting arrangement for the fiber output. The results in Figure 9 show improved fiber efficiencies of 16.6% at 10 kV to 12.0% at 40 kV for uncoated MOF with an air gap as shown in Figure 4. These values exceed the maximum theoretical light output of anthracene filled MOF.

The maximum theoretical output of anthracene filled MOF is based on work from liquid filled fiber where anthracene, using an effective refractive index of 1.62, is used instead of liquid [20]. A calculation of the photon capture probably in anthracene, with the quartz (fused silica) MOF acting as a cladding, is ~5% using Equations (2), (4), and (5) of reference [20]. This capture probability and representation of the anthracene in the MOF as bundle of optical fibers, each transmitting light through total internal reflection, provides the theoretical maximum output shown in Figure 9. Since the quartz MOF acts as a cladding, any coating has no effect on the theoretical maximum light output.

The improved X-ray efficiency of anthracene MOF occurs when 420 nm wavelength scintillation light travels through the air filled MOF quartz as shown in Figure 4. With ~5% of the scintillation light theoretically captured in anthracene, most (~95%) must travel through the quartz MOF. The capture of scintillation light through the quartz MOF allows for the improved efficiency. This improved efficiency, which involves complex three-dimensional optics, was observed for uncoated anthracene MOF. Coatings were applied and the resulting efficiency measured to aid in understanding the optics and the effect of coatings.

The use of aluminum and PFTE sputtered coatings was motivated to investigate the optical behavior of a highly reflective thin film from aluminum and a lower index of refraction thin film from PTFE, respectively. The effect of coatings with both an air gap and filled fiber shows low efficiencies, near that of uncoated filled fiber in Figure 9. Both coatings improve handling of the fibers.

Considerations in the sputtering of aluminum and PTFE were the vacuum and UV light effects on the anthracene. It was observed that anthracene fibers removed from the quartz and exposed to a 30 keV SEM electron beam ceased fluorescing when exposed to UV light. However, anthracene continued to fluoresce after sputter coating with both aluminum and PTFE.

Based on the experimental data in Figure 9, the improved efficiency of anthracene fiber with the air gap and no coating is a combination of several factors: the anthracene crystal quality, the air gap formed during anthracene solidification, and uncoated (bare) quartz. Although minor imperfections in the anthracene are found in all fibers, inspection of the fiber using visible and UV light must show that the fibers are free from voids and flaws shown in Figure 3 and an air gap exists between the light emitting end of the fiber and the anthracene as shown in Figure 4. These conditions must exist for a significant increase in the light output from the fiber. For example, whenever anthracene is grown in the MOF the beginning segment always has voids and must be removed; the opposite end has an air gap. These conditions provide an increase in efficiency of approximately 1300% at 40 kV and over 1600% at 10 kV compared to previous work as shown in Figure 9 [28].

## 4. Conclusions

Anthracene fibers are successfully grown from the melt in quartz MOF using a modified Bridgman-Stockbarger method. Void free fibers approximately 12 cm in length are grown consistently. Microstructured anthracene crystal growth conditions vary significantly from those of bulk growth. At temperatures of 230 and 70 °C for the melt and freeze zones, respectively, a growth rate of 25 mm/h is achieved. The Raman spectra shows that the anthracene fiber is a high purity molecular crystal; the XRD data shows the single crystal anthracene fiber has a (100) crystal orientation along the growth direction. Higher photon collection efficiency fibers are obtained by including the air gap formed during growth solidification and having no coating on the MOF quartz.
